# Risk Adjustment for Inter-Hospital Comparison of Caesarean Delivery Rates in Low-Risk Deliveries

**DOI:** 10.1371/journal.pone.0028060

**Published:** 2011-11-23

**Authors:** Elisa Stivanello, Paola Rucci, Elisa Carretta, Giulia Pieri, Chiara Seghieri, Sabina Nuti, Eugene Declercq, Martina Taglioni, Maria Pia Fantini

**Affiliations:** 1 Department of Medicine and Public Health – Alma Mater Studiorum University of Bologna, Bologna, Italy; 2 Laboratorio MeS, Scuola Superiore Sant'Anna, Pisa, Italy; 3 Department of Community Health Sciences, Boston University School of Public Health, Boston, Massachusetts, United States of America; 4 Medical Directorate, Azienda Ospedaliero-Universitaria di Bologna, Bologna, Italy; Hospital Clinic-University of Barcelona, Spain

## Abstract

**Background:**

Caesarean delivery (CD) rates have been frequently used as quality measures for maternity service comparisons. More recently, primary CD rates (CD in women without previous CD) or CD rates within selected categories such as nulliparous, term, cephalic singleton deliveries (NTCS) have been used. The objective of this study is to determine the extent to which risk adjustment for clinical and socio-demographic variables is needed for inter-hospital comparisons of CD rates in women without previous CD and in NTCS deliveries.

**Methods:**

Hospital discharge records of women who delivered in Emilia-Romagna Region (Italy) from January, 2007 to June 2009 and in Tuscany Region for year 2009 were linked with birth certificates. Adjusted RRs of CD in women without a previous Caesarean and NTCS were estimated using Poisson regression. Percentage differences in RR before and after adjustment were calculated and hospital rankings, based on crude and adjusted RRs, were examined.

**Results:**

Adjusted RR differed substantially from crude RR in women without a previous Caesarean and only marginally in NTCS group. Hospital ranking was markedly affected by adjustment in women without a previous CD, but less in NTCS.

**Conclusion:**

Risk adjustment is warranted for inter-hospital comparisons of primary CD rates but not for NTCS CD rates. Crude NTCS CD rates are a reliable estimate of adjusted NTCS CD.

## Introduction

The Caesarean delivery (CD) rate continues to rise in many countries worldwide even though this increase does not appear to be associated with improvement in maternal and perinatal mortality or morbidity [1]. Several studies suggested the benefit of multifaceted intervention, based on audit and detailed feedback activities, in improving clinical practice and effectively and safely reducing unnecessary CD [2], [3]. The most frequently used quality indicator to evaluate and compare maternal services is the overall CD rate [4]–[7]. However, recently this measure has been questioned and other measures have been introduced for audit activities and inter-hospital comparison [8]. Because of the existing controversy about safety of vaginal birth after a previous CD, several studies have recommended focusing quality efforts on primary CD rates (CD in women without previous CD) [4], [8]–[11]. Furthermore, based on evidence suggesting that non-vertex and multiple births may have better outcomes with CD [12], [13], some authors omitted these categories from the calculation of CD rates and examined only nulliparous, term, cephalic singleton deliveries (NTCS) [4], [9], [11], [13], [14]. This group accounts for a large proportion of CD and includes potentially lower-risk pregnancies [14]. In addition, NTCS is a group where efforts to reduce CD rates would lessen the need for repeat CD in subsequent pregnancies. Nevertheless, socio-demographic characteristics and/or clinical risk factors for CD might vary across hospitals. The use of unadjusted CD rates has been questioned and case-mix adjustment has been recommended for audit and inter-hospital comparisons of overall and primary CD rates [5], [6], [8], [15]–[17], but evidence about the need to use adjusted models for comparison of NTCS CD rates is limited [9], [14].

The aim of this study is to determine the extent to which adjustment for clinical and socio-demographic variables of the mother and the foetus enhances inter-hospital comparisons of primary and NTCS CD rates.

## Methods

For the purpose of this study, anonymised routine data obtained through record linkage of birth certificates and hospital discharge records of Emilia Romagna and Tuscany Regions (Italy) were used. The Emilia Romagna Region has 4.4 million inhabitants and approximately 40,000 births per year in 31 birth units. Tuscany Region has 3.7 million inhabitants and about 28,000 births per year in 34 birth units.

The study cohort includes women who delivered in public and private accredited Emilia Romagna birth units from January 1, 2007 to June 30, 2009 and in Tuscany birth units from January 2009 to December 2009. Hospital discharge records were identified using Disease Related Groups (DRGs) 370-375 or ICD-9 CM codes in primary or secondary diagnosis (V27xx or 640.xy-676.xy, where x = 0,…,9 and y = 1 or 2) or intervention codes (72.x, 73.2, 73.5, 73.6, 73.8, 73.9, 74.0, 74.1, 74.2, 74.4, 74.99). We excluded the following: 1) mothers under 11 and over 55 years of age, 2) mothers discharged from hospitals without an operating room or small hospitals (<150 deliveries per year), 3) mothers having hospital discharge records including intrauterine death (ICD-9 CM code 656.4) and still births (ICD-9 CM code V27.1, V27.4, V27.7).

We used birth certificates to identify parity, gestational age, plurality and presentation. We then selected two groups:

- Women without a previous CD (ICD-9 CM diagnosis code 654.2x).
- NTCS – nulliparous, term, cephalic, singleton deliveries (defined by birth certificate information).

Maternal age, educational level, citizenship and marital status were retrieved from birth certificates. Information on maternal and foetal clinical risk factors was retrieved from hospital discharge records (index and previous hospitalisations) and/or birth certificates. This included: HIV, diabetes, hypertension, thyroid diseases, other severe comorbidities, genital herpes, substance abuse, eclampsia or pre-eclampsia, placenta praevia or abruption or ante partum hemorrhage, cephalopelvic disproportion, RH isoimmunisation, polihydramnios, oligohydramnios, premature rupture of membranes of the amnios, other problems of the amnios, cord prolapse, abortion threads, in vitro fertilization, or supervision of high risk pregnancy, intra-uterine growth retardation, foetal weight, foetal malformation. We also examined pregnancy length, multiple births and presentation other than vertex as additional potential independent risk factors for primary CD, but not for NTCS.

We excluded foetal distress and uterine dystocia as potential risk factors, because these might be a reason for an ex-post justification of the CD [16]–[18].

The study was carried out in compliance with the Italian law on privacy (Art. 20–21, DL 196/2003) and the regulations of the Regional Health Authorities of Emilia-Romagna and Tuscany Regions on data management. Data were anonymized at the regional statistical office where each patient was assigned a unique identifier that is the same for all administrative databases. This identifier does not allow to trace the patient's identity and other sensitive data. When anonymized administrative data are used to inform health care planning activities, the study is exempt from notification to the Ethics Committee and no specific written consent is needed to use patient information stored in the hospital databases.

### Statistical analysis

Primary and NTCS CD rates and their coefficient of variation (CV) by hospital were estimated. The relative risk of CD for each hospital was estimated in women without a previous CD and in the NTCS group using Poisson regression models, to control for demographic and clinical confounders [19]. Demographic and clinical risk factors of CD to be included in the risk adjustment models were selected using a backward stepwise selection procedure. This procedure includes initially all factors identified as potential predictors of CD and then removes factors not associated with CD at p<0.05. The reference category for the calculation of the RR was identified using a recursive procedure set up by the P.Re.Val.E. Project [20]. This procedure enables identification of a homogeneous subset of hospitals with the lowest adjusted risk of CD. The procedure was replicated for each of the two study groups. Crude and adjusted RRs were used to rank hospitals and the percentage difference between crude and adjusted RR was calculated. A percentage difference greater than 10% and a change in ranking >3 were considered as relevant [21]. The correlation between crude and adjusted CD rates was assessed using Spearman's correlation coefficient.

C-index and AIC were used to evaluate the goodness of fit of the models. Specifically, the C-index was used to assess how well the model discriminates between women with and without a CD. The area under the curve ranges from 0.50 (no ability to discriminate) to 1 (perfect discrimination). the AIC (Akaike Information Criterion) is a measure that combines fit and complexity of the model. Lower values indicate a better fit of the model taking complexity into account.

All statistical analyses were performed using Stata 10 (College Station, Texas 77845, USA).

## Results

During the study period, in the Emilia Romagna Region, in 24 hospitals there was a total of 98,913 deliveries, of which 87,849 had no previous CD and 46,179 were NTCS. The overall CD rate was 30.3% (range 19.2–53.9%, Coefficient of variation-CV: 22.6); the primary CD rate was 22.4%, (range 13.3–40.2%, CV: 25.3) and the NTCS CD rate was 23.4% (range 12.7–42.5%, CV: 27.1). The NTCS CD rate contributed 37.5% of overall CD rate (range 26–46%, CV: 13.6).

Table S1 reports crude and adjusted primary CD rates and crude and adjusted RRs for each hospital compared to the reference category. Variables included in the final models and goodness of fit indices are listed as a note to the table. In the Emilia Romagna Region, the primary CD rate in the reference category, including five hospitals, was 15.0%. In the other hospitals, crude CD rates ranged from 16.8% (Hospital M) to 40.2% (Hospital E) while after adjustment, rates ranged from 16.9% (Hospital F) to 31.3% (Hospital E). Compared with the reference category, the adjusted RRs ranged from 1.13 (Hospital F) to 2.09. In women without previous CD, 9/24 hospitals showed a >10% variation between crude and adjusted RRs, and 8 out of those 9 hospitals had their risk reduced compared with the reference category. There was no significant difference on number of deliveries and the presence/absence of training programs between hospital that changed and those that did not change, both for primary CD and NTCS (data not shown). Of the 15 hospitals showing a ≤10% difference between crude and adjusted RRs, 5 had their risk reduced and 10 increased

Table S2 reports the results for the NTCS group. The CD rate in the reference category, including the same five hospitals, was 14.5%, the highest unadjusted rate in Emilia Romagna Region was 42.6% (Hospital E) and the highest adjusted rate was 38.2%. In the NTCS sample, a>10% difference between crude and adjusted RRs was found in 2 hospitals, and in both cases adjusted RRs were lower than crude RR. Six hospitals in the NTCS group had adjusted RRs more than twice as high as those of the reference group.

As for hospital ranking using crude and adjusted RR of primary CD, in women without previous CD, 23 hospitals had their rank changed after adjustment: four hospitals differed 1 rank, seven differed 2–3 ranks, twelve differed 4–11 ranks. In the NTCS group, ranking based on adjusted measures varied for 13 hospitals: seven differed 1 rank, five differed 2–3 ranks, one differed 7 ranks.

Spearman's correlation coefficients between crude NTCS and adjusted primary CD rates and between crude and adjusted NTCS CD rates were r = 0.81 and r = 0.97.

In the year 2009, in the Tuscany Region, of the 29,438 deliveries, 26,851 were deliveries without a previous CD across 26 hospitals and 12,433 met the NTCS inclusion criteria across 25 hospitals One hospital was excluded in the analyses of the NTCS study group because of incomplete data on parity. In women without a previous CD, the reference category included one hospital with a CD rate of 9.2%. After adjusting for clinical and socio-demographic variables, 10 hospitals showed a difference in RR greater than 10% and twenty-two hospitals changed their rank: five hospitals differed 1 rank, fourteen differed 2–3 ranks, three differed 4–10 ranks. In the NTCS group the reference category included three hospitals with an average rate of 11.1%. After adjusting for clinical and socio-demographic variables, a difference in adjusted RR higher than 10% was found in 5 hospitals, and all the adjusted RR were lower than the crude RR. Ranking based on adjusted measures varied for 14 hospitals: nine differed 1 rank, five differed 2–3 ranks.

Spearman's correlation coefficient between crude and adjusted primary CD rates was r = 0.92 and between crude and adjusted NTCS CD rates was r = 0.98.

In both regions, a sensitivity analysis was carried out to explore the role of cephalopelvic disproportion as potential confounder. Hospital adjusted RRs (for primary and NTCS CD) were calculated after excluding this variable from the model. No substantial differences were observed between adjusted RRs obtained with and without this variable (data available on request).

In a secondary sensitivity analysis we excluded all deliveries with a clinical condition resulting in appropriate CD (genital HSV, HIV, cord prolapse, malpresentation) and calculated adjusted RR in each cohort of the two regions. In the cohort of primary CD, a >10% difference between crude and adjusted RR was found in 15 hospitals in Emilia-Romagna and 15 in Tuscany. In the NTCS group, a >10% difference between crude and adjusted RR was found in 4 hospital in Emilia-Romagna and 8 in Tuscany.

### Identification of outliers

In the present study, interhospital comparison is based on the use of a reference group of hospitals with the best performance. Outliers are therefore those with the highest difference from the reference group, after adjusting for case mix.

**Figure 1:**
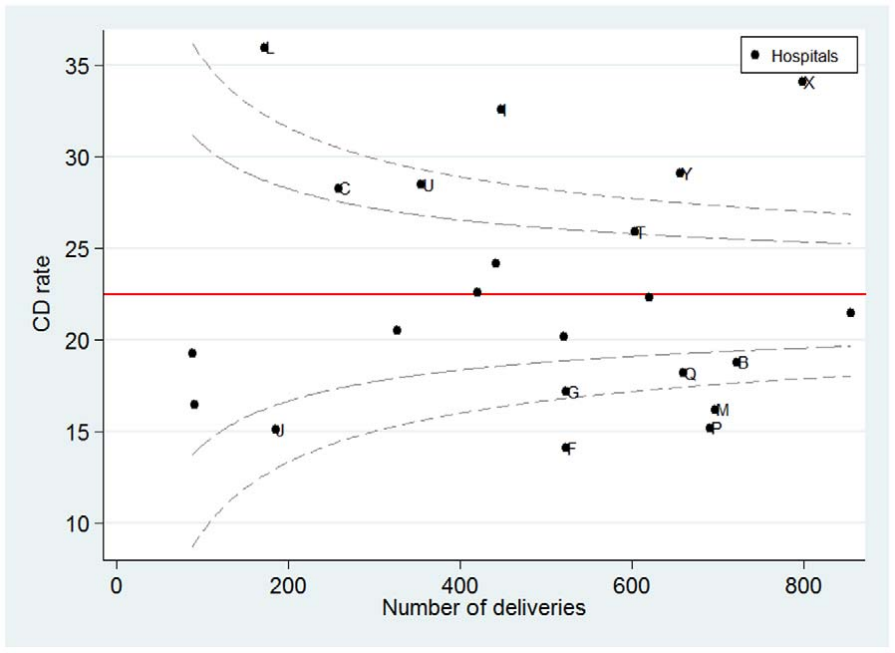
Funnel plot showing the relationship between the number of deliveries and NTCS CD rates in the hospitals of Tuscany. Dashed lines indicate the 95% and 99.8% CI for the CD rates.

Alternative methods for interhospital comparison using the regional mean as the reference group allow identification of outliers with adjusted CD rates lower or higher than the regional mean. For example, using the funnel plot as the graphical representation of adjusted CD rates vs. the number of deliveries, we found that, in Tuscany, 5 hospitals had adjusted CD rates exceeding the upper level of the 99.8% confidence interval of the mean. Of note, in the NTCS group, the outlier hospitals with significantly higher unadjusted CD rates than the regional mean (Figure 1) were the same as those in the primary CD group.

## Discussion

Our results indicate substantial inter-hospital variations in both primary and NTCS CD rates. NTCS CD contributed more than one third of the overall CD rate, and more than one half of primary CD, in line with other studies [4], [9], [14], [22], [23]. The main strength of our study consists in the relatively large number of deliveries from 50 hospitals of two different regions.

We found that the use of risk adjustment procedures had a different importance when comparing hospitals in terms of primary or NTCS CD rates. When the focus was on primary CD rates, adjustment for clinical and socio-demographic factors affected the RRs in several hospitals and led to a substantial variation in their ranking. On the contrary, in the NTCS group, risk adjustment led to a lower change in RRs and more limited variation in the hospital ranking. Morevoer, crude NTCS CD rates highly correlated with adjusted NTCS CD rates. These findings were consistent between the two Italian regions studied.

In order to explain the modest difference between crude and adjusted RR in NTCS compared to primary CD, it must be recalled that the NTCS groups is homogeneous for gestational age, presentation, singleton birth and nulliparity by definition.

Therefore our results suggest that the NTCS cohort excludes frequent conditions that may be unevenly distributed across hospitals. All other factors we controlled for in this cohort, including mother's comorbidities or demographic variables and other less frequent obstetric conditions did not confound substantially the results.

Sensitivity analyses excluding from the primary and NTCS cohorts factors for which the CD is appropriate (HIV, malpresentation, cord prolapse and genital herpes) did not reduce interhospital variability and the discrepancy between crude and adjusted rates.

Lastly, we found that outlier hospitals with high CD rates can be consistently identified using two methods for interhospital comparison that use best performing hospitals or the regional mean as the reference group. Furthermore, outlier hospitals in the primary CD and NTCS cohort are the same.

Inter-hospital variation of CD rates persisted after adjustment for clinical and socio-demographic variables in all women without a previous CD and in the NCTS group, suggesting that variablility should be ascribed to other unexplored clinical or non medical factors, including organization in the birth units, staff attitudes, cultural backgrounds and women's choice [24]–[26].

Few studies have addressed the relevance of applying risk adjustment procedures to compare NTCS CD rates across hospitals. Main et al [4] reported that after controlling for age, some hospitals showed small differences in CD rates, but 5 out of 20 with age-skewed populations had a 2.5–5% reduction in CD rates. They concluded that age-adjusted NTCS CD rates are a promising quality measure. Coonrod et al. [14] using NTCS CD rates, reported that 31/40 hospitals retained their original ranking before and after adjustment for clinical factors, but 4 changed their status from an outlier to an average-risk hospital. These authors argued that hospital quality assessment programs may require risk adjusted NTCS CD rates [14].

Our results should be interpreted keeping in mind some limitations. First, we used administrative databases, consistent with studies monitoring CD rates for quality of care assessment. Multiple issues regarding the validity of administrative data remain largely unexplored [27]. Problems in accuracy, completeness and quality might differ from hospital to hospital, errors in coding may occur and omissions of ICD codes identifying risk factors may be more likely in the group without a CD. However, in Emilia Romagna and Tuscany Region, the administrative databases proved to have a high degree of completeness and quality and have already been used in studies using the same data sources [28]. Second, other risk factors for CD such as body mass index and gestational weight gain [29] could not be included in the risk adjustment model because information on height and weight is not recorded in our databases. Nevertheless, to date administrative databases continue to be the most viable solution to study temporal or geographical variations at national or regional level, healthcare outcomes and quality of care [7]. Lastly, since the impact of variables used for risk adjustment may vary across populations, generalization of our results should be done with caution.

Taking into account differences in case mix across hospitals is fair and appropriate, however, we submit that crude NTCS measures can be reliably used for inter-hospital comparison. NTCS CD rates are easily retrieved from birth certificate or vital statistics systems in many countries, thereby avoiding linkage procedures with other datasets that contain more detailed clinical information. Since in our population adjusted and crude NTCS CD rates lead to consistent hospital rankings and crude NTCS rates are highly correlated with adjusted CD rates, the contention that differences in case mix are responsible for differences in CD rates could be minimized. In addition, by using this measure, a substantial proportion of primary CD are captured.

US and European studies have shown that providing feedback to caregivers on their own performances relative to their peers can significantly reduce CD rates [30], [31]. Efforts to reduce primary CD rates will, in turn, have the added benefit of reducing the total number of repeated CD. Our study contributes to identifying an efficient way to make inter-hospital comparisons using routinely collected data.

In conclusion, our findings show that risk adjustment is warranted for inter-hospital comparisons of primary CD rates, but is less compelling for NTCS CD rates. Inter-hospital comparison of NTCS CD rates has the potential to identify overuse of CD in low-risk primigravidas and to inform attempts to reduce hospital CD rates.

## Supporting Information

Table S1 **Crude and adjusted primary CD rates and RR by hospital in Emilia Romagna and Tuscany Regions.**
(DOC)

Table S2 **Crude and adjusted NTCS CD rates and RR by Hospital in Emilia Romagna and Tuscany Regions.**
(DOC)
