# Supplementary material for: Risk Adjustment for Inter-Hospital Comparison of Caesarean Delivery Rates in Low-Risk Deliveries
Source: PLoS One. 2011 Nov 23;6(11):e28060. doi: 10.1371/journal.pone.0028060 (PMC3223220; doi:10.1371/journal.pone.0028060)
Supplement: Table S1 — Crude and adjusted primary CD rates and RR by hospital in Emilia Romagna and Tuscany Regions. (DOC) [file pone.0028060.s001.doc]

**Table 1. Crude and adjusted primary CD rates and RR by h**ospital in Emilia Romagna and Tuscany Regions.

|  | Emilia Romagna Region | | | | | | |  |  | Tuscany Region | | | | | | |
| --- | --- | --- | --- | --- | --- | --- | --- | --- | --- | --- | --- | --- | --- | --- | --- | --- |
| Hospital | Number of deliveries | crude CD rate (%) | adj* CD rate (%) | crude RR | adj* RR | *P* | % difference |  | Hospital | Number of deliveries | crude CD rate (%) | adj* CD rate (%) | crude RR | adj* RR | *P* | % difference |
| A | 2321 | 23.8 | 25.0 | 1.59 | 1.67 | <0.001 | 5.1 |  | A | 1,169 | 17.5 | 16.1 | 1.90 | 1.75 | <0.001 | -7.9 |
| B^ | 3288 | 24.1 | 18.8 | 1.60 | 1.25 | <0.001 | -22.1 |  | B | 157 | 15.3 | 15.8 | 1.66 | 1.71 | 0.001 | 3.3 |
| C | 483 | 27.5 | 29.2 | 1.84 | 1.95 | <0.001 | 6.0 |  | C | 451 | 25.5 | 20.2 | 2.77 | 2.19 | <0.001 | -20.9 |
| D | 510 | 26.7 | 28.9 | 1.78 | 1.93 | <0.001 | 8.4 |  | D | 791 | 19.1 | 16.8 | 2.07 | 1.83 | <0.001 | -11.8 |
| E^ | 784 | 40.2 | 31.3 | 2.68 | 2.09 | <0.001 | -22.1 |  | E | 1,120 | 19.3 | 19.3 | 2.09 | 2.10 | <0.001 | 0.1 |
| F | 2037 | 17.2 | 16.9 | 1.15 | 1.13 | 0.01 | -1.5 |  | F | 971 | 13.2 | 12.6 | 1.43 | 1.37 | 0.001 | -4.6 |
| G | 1531 | 20.6 | 21.7 | 1.38 | 1.45 | <0.001 | 5.2 |  | G | 1,042 | 16.2 | 15.1 | 1.76 | 1.64 | <0.001 | -6.8 |
| H | 1710 | 24.6 | 26.5 | 1.64 | 1.77 | <0.001 | 7.6 |  | H | 745 | 23.1 | 21.2 | 2.51 | 2.31 | <0.001 | -8.0 |
| I | 3456 | 21.6 | 22.4 | 1.44 | 1.50 | <0.001 | 4.2 |  | I | 813 | 28.7 | 26.0 | 3.11 | 2.82 | <0.001 | -9.4 |
| J | 1871 | 18.1 | 19.8 | 1.21 | 1.32 | <0.001 | 9.4 |  | J | 349 | 14.9 | 14.8 | 1.62 | 1.60 | <0.001 | -0.9 |
| K | 2503 | 23.6 | 21.8 | 1.57 | 1.45 | <0.001 | -7.9 |  | K | 189 | 16.9 | 15.0 | 1.84 | 1.63 | 0.001 | -11.3 |
| L | 6415 | 22.0 | 19.1 | 1.47 | 1.27 | <0.001 | -13.6 |  | L | 282 | 29.1 | 27.0 | 3.16 | 2.93 | <0.001 | -7.3 |
| M | 1367 | 16.8 | 17.4 | 1.12 | 1.16 | 0.01 | 3.2 |  | M | 1,292 | 16.5 | 13.5 | 1.79 | 1.47 | <0.001 | -18.0 |
| N | 1917 | 20.7 | 20.6 | 1.38 | 1.37 | <0.001 | -0.7 |  | N | 1,188 | 20.4 | 14.9 | 2.21 | 1.61 | <0.001 | -27.1 |
| O | 1292 | 22.8 | 20.4 | 1.52 | 1.36 | <0.001 | -10.5 |  | O | 603 | 14.9 | 14.9 | 1.62 | 1.62 | <0.001 | 0.0 |
| P^ | 5598 | 30.4 | 24.6 | 2.03 | 1.64 | <0.001 | -19.1 |  | P | 1,616 | 17.0 | 16.9 | 1.84 | 1.84 | <0.001 | -0.1 |
| Q | 3008 | 26.9 | 26.7 | 1.80 | 1.78 | <0.001 | -0.9 |  | Q | 1,598 | 20.7 | 19.7 | 2.24 | 2.14 | <0.001 | -4.6 |
| R | 7248 | 24.4 | 22.4 | 1.63 | 1.49 | <0.001 | -8.4 |  | R | 927 | 18.6 | 18.8 | 2.02 | 2.04 | <0.001 | 1.2 |
| S | 3277 | 24.4 | 21.8 | 1.63 | 1.46 | <0.001 | -10.5 |  | S | 527 | 20.5 | 19.1 | 2.23 | 2.07 | <0.001 | -7.0 |
| T | 6212 | 21.5 | 22.1 | 1.44 | 1.47 | <0.001 | 2.3 |  | T | 1,123 | 24.4 | 20.0 | 2.65 | 2.17 | <0.001 | -18.2 |
| U | 5415 | 19.9 | 17.5 | 1.33 | 1.17 | <0.001 | -12.1 |  | U | 661 | 24.4 | 20.3 | 2.65 | 2.20 | <0.001 | -16.8 |
| W^ | 7961 | 28.3 | 24.8 | 1.89 | 1.66 | <0.001 | -12.1 |  | W | 1,680 | 18.7 | 19.0 | 2.03 | 2.07 | <0.001 | 1.8 |
| X | 2372 | 21.4 | 22.1 | 1.43 | 1.48 | <0.001 | 3.7 |  | X^ | 1,456 | 34.5 | 23.3 | 3.75 | 2.53 | <0.001 | -32.6 |
| Y | 1560 | 22.6 | 25.1 | 1.51 | 1.67 | <0.001 | 10.8 |  | Y^ | 1,185 | 30.8 | 21.6 | 3.35 | 2.35 | <0.001 | -29.8 |
|  |  |  |  |  |  |  |  |  | Z^ | 2,353 | 29.7 | 21.8 | 3.22 | 2.36 | <0.001 | -26.6 |
| REF | 13713 | 15.0 |  | 1.00 | 1.00 |  |  |  | REF | 2563 | 9.2 |  | 1.00 | 1.00 |  |  |

| *adjusted for: age, citizenship, marital status, educational level, severe comorbidities, HIV, hypertension, diabetes, lung problems, eclampsia/preeclampsia, isoimmunization, abruptio or placenta previa or ante-partum hemorrhage, fetal or maternal disproportion, isoimmunization, polihydramnios, oligohydramnios, premature rupture of membranes of the amnios, other problems of the amnios, high risk pregnancies (abortion threads, assisted fecundation), fetal weight, fetal malformation, intrauterine growth retardation, cord prolapse, pregnancy length, multiple births, presentation other than vertex  ^teaching hospital  **Goodness of fit indices**  c-index 0.77; Log-likelihood: null model -46540.98, model with covariates -34671.18 ; AIC=69422.37 |  | *adjusted for: age, citizenship, marital status, severe comorbidities, hypertension, lung problems, abruptio or placenta previa or ante-partum hemorrhage, fetal or maternal disproportion, polihydramnios, oligohydramnios, premature rupture of membranes of the amnios, other problems of the amnios, fetal malformation, intrauterine growth retardation, cord prolapse, pregnancy length, multiple births, presentation other than vertex, high risk pregnancies (abortion threads, assisted fecundation).  ^teaching hospital  **Goodness of fit indices**  c-index 0.79 ; Log-likelihood: null model -13546.24, model with covariates -9727.492 ; AIC=19514.98 |
| --- | --- | --- |

CD: caesarean delivery; RR: relative risk.
