# Supplementary material for: Risk Adjustment for Inter-Hospital Comparison of Caesarean Delivery Rates in Low-Risk Deliveries
Source: PLoS One. 2011 Nov 23;6(11):e28060. doi: 10.1371/journal.pone.0028060 (PMC3223220; doi:10.1371/journal.pone.0028060)
Supplement: Table S2 — Crude and adjusted NTCS CD rates and RR by Hospital in Emilia Romagna and Tuscany Regions. (DOC) [file pone.0028060.s002.doc]

**Table 2. Crude and adjusted NTCS CD rates and RR by Hospital in Emilia Romagna and Tuscany Regions.**

|  | Emilia Romagna Region | | | | | | | |  |  | | Tuscany Region | | | | | | |
| --- | --- | --- | --- | --- | --- | --- | --- | --- | --- | --- | --- | --- | --- | --- | --- | --- | --- | --- |
| Hospital | | Number of deliveries | crude CD rate (%) | *adj CD rate (%) | crude RR | *adj RR | *P* | % difference |  | Hospital | Number of deliveries | | crude CD rate (%) | *adj CD rate (%) | crude RR | *adj RR | *P* | % difference |
| A | | 1288 | 26.5 | 25.8 | 1.81 | 1.77 | <0.001 | -2.5 |  | A | 722 | | 18.8 | 18.9 | 1.70 | 1.70 | <0.001 | 0.1 |
| B^ | | 1821 | 20.3 | 18.8 | 1.39 | 1.29 | <0.001 | -7.4 |  | B | 88 | | 19.3 | 19.5 | 1.75 | 1.76 | 0.01 | 0.7 |
| C | | 236 | 30.9 | 29.3 | 2.12 | 2.01 | <0.001 | -5.2 |  | C | 258 | | 28.3 | 23.4 | 2.56 | 2.11 | <0.001 | -17.4 |
| D | | 279 | 34.8 | 32.7 | 2.38 | 2.25 | <0.001 | -5.6 |  | D | 420 | | 22.6 | 22.6 | 2.04 | 2.04 | <0.001 | -0.3 |
| E^ | | 463 | 42.6 | 38.2 | 2.92 | 2.63 | <0.001 | -9.8 |  | E | 620 | | 22.3 | 22.3 | 2.01 | 2.02 | <0.001 | 0.3 |
| F | | 1100 | 20.1 | 18.6 | 1.38 | 1.28 | <0.001 | -7.1 |  | F | 523 | | 14.1 | 13.9 | 1.28 | 1.25 | 0.08 | -1.9 |
| G | | 819 | 23.0 | 22.7 | 1.57 | 1.56 | <0.001 | -0.9 |  | G | 523 | | 17.2 | 18.4 | 1.55 | 1.66 | <0.001 | 6.8 |
| H | | 921 | 30.4 | 29.9 | 2.08 | 2.05 | <0.001 | -1.6 |  | H | 442 | | 24.2 | 24.0 | 2.19 | 2.16 | <0.001 | -1.0 |
| I | | 1934 | 23.9 | 23.8 | 1.64 | 1.64 | <0.001 | 0.1 |  | I | 448 | | 32.6 | 34.6 | 2.94 | 3.12 | <0.001 | 6.1 |
| J | | 932 | 20.4 | 21.5 | 1.40 | 1.48 | <0.001 | 5.9 |  | J | 185 | | 15.1 | 15.8 | 1.37 | 1.42 | 0.05 | 4.2 |
| K | | 1329 | 27.3 | 25.8 | 1.87 | 1.78 | <0.001 | -4.9 |  | K | 91 | | 16.5 | 16.5 | 1.49 | 1.49 | 0.10 | 0.1 |
| L | | 3521 | 21.9 | 19.2 | 1.50 | 1.32 | <0.001 | -12.2 |  | L | 172 | | 36.0 | 32.0 | 3.26 | 2.89 | <0.001 | -11.2 |
| M | | 772 | 17.5 | 17.2 | 1.20 | 1.18 | 0.03 | -1.6 |  | M | 696 | | 16.2 | 15.0 | 1.47 | 1.35 | 0.01 | -7.8 |
| N | | 977 | 22.3 | 22.3 | 1.53 | 1.53 | <0.001 | 0.0 |  | P | 691 | | 15.2 | 15.8 | 1.37 | 1.42 | 0.00 | 3.8 |
| O | | 737 | 25.1 | 20.6 | 1.72 | 1.42 | <0.001 | -17.5 |  | Q | 659 | | 18.2 | 17.9 | 1.65 | 1.62 | <0.001 | -1.7 |
| P^ | | 2846 | 32.2 | 30.0 | 2.21 | 2.06 | <0.001 | -6.7 |  | R | 521 | | 20.2 | 19.6 | 1.82 | 1.77 | <0.001 | -2.8 |
| Q | | 1485 | 31.5 | 30.0 | 2.16 | 2.06 | <0.001 | -4.6 |  | S | 327 | | 20.5 | 21.5 | 1.85 | 1.94 | <0.001 | 4.8 |
| R | | 3506 | 25.2 | 24.4 | 1.73 | 1.68 | <0.001 | -2.8 |  | T | 603 | | 25.9 | 23.2 | 2.34 | 2.09 | <0.001 | -10.4 |
| S | | 1751 | 23.9 | 22.3 | 1.64 | 1.53 | <0.001 | -6.5 |  | U | 354 | | 28.5 | 24.7 | 2.58 | 2.23 | <0.001 | -13.5 |
| T | | 3657 | 20.7 | 21.4 | 1.42 | 1.47 | <0.001 | 3.5 |  | W | 855 | | 21.5 | 21.3 | 1.94 | 1.92 | <0.001 | -1.2 |
| U | | 2557 | 17.6 | 17.3 | 1.20 | 1.19 | <0.001 | -1.1 |  | X^ | 798 | | 34.1 | 30.8 | 3.08 | 2.78 | <0.001 | -9.7 |
| W^ | | 4395 | 29.8 | 27.6 | 2.04 | 1.90 | <0.001 | -7.1 |  | Y^ | 656 | | 29.1 | 23.4 | 2.63 | 2.11 | <0.001 | -19.7 |
| X | | 1258 | 23.5 | 24.1 | 1.61 | 1.66 | <0.001 | 3.3 |  |  |  | |  |  |  |  |  |  |
| Y | | 781 | 27.3 | 28.3 | 1.87 | 1.95 | <0.001 | 4.3 |  |  |  | |  |  |  |  |  |  |
| REF | | 6814 | 14.5 |  | 1.00 | 1.00 |  |  |  | REF | 2013 | | 11.1 |  | 1.00 | 1.00 |  |  |

| *adjusted for: age, citizenship, marital status, educational level, severe comorbidities, HIV, hypertension, diabetes, lung problems, substance abuse, eclampsia/preeclampsia, abruptio or placenta previa or ante-partum hemorrhage, fetal or maternal disproportion, isoimmunization, polihydramnios, oligohydramnios, premature rupture of membranes of the amnios, other problem of the amnios, high risk pregnancies (abortion threads, assisted fecundation), fetal weight, fetal malformation, intrauterine growth retardation.  ^teaching hospital  **Goodness of fit indices**  c-index 0.68  log-likelihood: null model -25110.74 , model with covariates 22783.23  AIC= 45636.47 |  | *adjusted for: age, maternal status, educational leval, severe comorbidities, diabetes, hypertension, eclampsia/preeclampsia, abruptio or placenta previa or ante-partum hemorrhage, fetal or maternal disproportion, polihydramnios, oligohydramnios, other problem of the amnios, high risk pregnancies (abortion threads, assisted fecundation), previous still dead/abortion, fetal weight, intrauterine growth retardation  ^teaching hospital  **Goodness of fit indices**  c-index 0.68  log-likelihood: null model -6343.456 , model with covariates -5652.888  AIC= 11353.78 |
| --- | --- | --- |

NTCS, nulliparous, term, cephalic, singleton deliveries; CD, caesarean delivery; RR, relative risk.
